# Supplementary material for: How European Research Projects Can Support Vaccination Strategies: The Case of the ORCHESTRA Project for SARS-CoV-2
Source: Vaccines (Basel). 2023 Aug 14;11(8):1361. doi: 10.3390/vaccines11081361 (PMC10459328; doi:10.3390/vaccines11081361)
Supplement: Supplementary file 1 [file vaccines-11-01361-s001.zip › vaccines-2510017-supplementary.pdf]

Supplementary material

“How European research projects can support vaccination strategies: the case of the ORCHESTRA project for SARS-CoV-2”

Anna Maria Azzini, Lorenzo Maria Canziani et al.

Index

Supplementary table 1: ORCHESTRA cohorts 1

List of abbreviations........ 3

Supplementary table 1: List of ORCHESTRA cohorts

**Main Partner (Cohort name)**	**Country**	**Number of subjects**	**Established before ORCHESTRA beginning**
**COVID-19 cohorts and long-term sequelae**			
INSERM (French COVID-19)	France	4489	Yes
UNIVR	Italy	2224	Yes
UNIBO	Italy	515	Yes
UMGC (COVID-HOME)	The Netherlands	190	Yes
UBA	Argentina	14	No
UHC (NAPKON/SÜP)	Germany	2479	Yes
SAS (SAS-HUVM)	Spain	1356	Yes
UMCG (COVID-19 ICU)	The Netherlands	397	Yes
FCRM	Congo	30	Yes
University of Tuebingen (VGCARE)	Vietnam	24	No
**Patient registries**			
RER-ASSR	Italy	1877841	Yes
REG VEN	Italy	450000	Yes
UHC (LEOSS)	Germany	12981	Yes
PENTA (PEDIANET)	Italy	16060	Yes
**General population cohorts**			
HMGU (GNC / NAKO)	Germany	205217	Yes
INSERM (EPICOV)	France	134391	Yes
UMCG (Lifelines)	The Netherlands	76503	Yes
ISGlobal (CONTENT)	Spain	20000	Yes
LMU (KoCo19)	Germany	6085	Yes
RER-ASSR (CoVstories)	Italy	2656	No
UNIVR (Sentinella)	Italy	2000	No
LIH (CON-VINCE)	Luxembourg	1865	Yes
PENTA (CASE cohort)	Italy	200	No
**Fragile population cohorts – people living with HIV**			
ICONA	Italy	5746	Yes
UNIBO (CONTRAST)	Italy	436	Yes
UNIVR	Italy	304	No

CERMEL	Gabon	300	No
CBCI	India	189	No
UBA	Argentina	87	No
UCD (All-Ireland Infectious Diseases Cohort)	Ireland	35	Yes
SAS	Spain	14	No
**Fragile population cohorts - solid organ transplant recipients**			
REG VEN	Italy	845	No
UNIBO (CONTRAST)	Italy	673	Yes
PENTA (Brazil_RETR COVID SOT)	Brazil	470	Yes
UNIVR	Italy	235	No
University of Padua	Italy	159	No
SAS	Spain	146	No
**Fragile populations cohorts - solid cancer**			
UNIVR	Italy	926	No
UNIBO (CONTRAST)	Italy	223	Yes
SAS	Spain	91	No
**Fragile population cohorts – hematological malignancy**			
UNIVR	Italy	370	No
UNIBO (CONTRAST)	Italy	35	Yes
SAS	Spain	32	No
**Fragile population cohorts - rheumatological patients**			
CBCI	India	136	No
SAS	Spain	32	No
**Fragile population cohorts - pregnancy and newborns**			
UMCG (ZIKAlliance SARS-CoV-2 substudy)	Netherlands, Germany, Venezuela, Perú, Colombia, Brazil	3111	No
**Fragile population cohorts - pregnancy**			
THSTI (GARBH-Ini)	India	140	Yes
**Fragile population cohort - children**			
PENTA (CASE – household)	Italy	1577	Yes
PENTA (EPICO-AEP)	Spain	1340	Yes
**Fragile populations cohorts – cystic fibrosis**			
UNIVR	Italy	247	No
**Fragile populations cohorts – Parkinson disease**			
LIH (NCER-PD)	Luxembourg	154	Yes
**Fragile populations cohorts - Hemodialysis**			
SAS	Spain	6	No
**Healthcare workers cohorts**			
AP-HP	France	80746	Yes
UNIVR	Italy	31909	Yes
UNIBO	Italy	27246	Yes
UNIOVI	Spain	8385	Yes
University of Bari	Italy	6196	Yes

LMU	Germany	3282	Yes
INSP	Romania	2698	Yes
Raph BB	Slovakia	1072	Yes
IDIAP JGol	Spain	855	Yes

List of abbreviations
AP-HP: Assistance Publique Hopitaux de Paris
CBCI: Catholics Bishops Conference of India
CERMEL: Centre de Recherche Medicales de Lambaréné
FCRM: Fondation Congolaise pour la Recherche Medicale
ICONA: ICONA Foundation
IDIAP JGol: Institut Universitari d'Investigació en Atenció Primària Jordi Gol
INSP: Institutul National De Sanatate Publica
INSERM: Institut National de la Sante et de la Recherche Medicale
ISGlobal: Fundacion Privada Instituto de Salud Global Barcelona
LIH: Luxembourg Institute of Health
LMU: Ludwig-Maximilians-Universitaet Muenchen
PENTA: PENTA foundation
RAPH BB: Regionalny Uras Verejneho Zdravotnictva so Sidlom v Banskej Bystrici
REG VEN: Veneto Region
RER-ASSR: Emilia-Romagna Region
SAS: Servicio Andaluz de Salud
SOT: Solid organ transplantation
THSTI: Translational Health Science and Technology Institute, Faridabad
UBA: University of Buenos Aires
UCD: University College Dublin
UHC: Klinikum der Universität zu Koeln
UMGC: Academisch Ziekenhuis Groningen
UNIBO: University of Bologna
UNIOVI: University of Oviedo
UNIVR: University of Verona
